# Hybrid convolution neural network with channel attention mechanism for sensor-based human activity recognition

**DOI:** 10.1038/s41598-023-39080-y

**Published:** 2023-07-26

**Authors:** Sakorn Mekruksavanich, Anuchit Jitpattanakul

**Affiliations:** 1 Department of Computer Engineering, School of Information and Communication Technology, University of Phayao, Phayao, 56000 Thailand; 2 Department of Mathematics, Faculty of Applied Science, King Mongkut’s University of Technology North Bangkok, Bangkok, 10800 Thailand; 3 Intelligent and Nonlinear Dynamic Innovations Research Center,Science and Technology Research Institute, King Mongkut’s University of Technology North Bangkok, Bangkok, 10800 Thailand

**Keywords:** Computer science, Information technology

## Abstract

In the field of machine intelligence and ubiquitous computing, there has been a growing interest in human activity recognition using wearable sensors. Over the past few decades, researchers have extensively explored learning-based methods to develop effective models for identifying human behaviors. Deep learning algorithms, known for their powerful feature extraction capabilities, have played a prominent role in this area. These algorithms can conveniently extract features that enable excellent recognition performance. However, many successful deep learning approaches have been built upon complex models with multiple hyperparameters. This paper examines the current research on human activity recognition using deep learning techniques and discusses appropriate recognition strategies. Initially, we employed multiple convolutional neural networks to determine an effective architecture for human activity recognition. Subsequently, we developed a hybrid convolutional neural network that incorporates a channel attention mechanism. This mechanism enables the network to capture deep spatio-temporal characteristics in a hierarchical manner and distinguish between different human movements in everyday life. Our investigations, using the UCI-HAR, WISDM, and IM-WSHA datasets, demonstrated that our proposed model, which includes cross-channel multi-size convolution transformations, outperformed previous deep learning architectures with accuracy rates of 98.92%, 98.80%, and 98.45% respectively. These results indicate that the suggested model surpasses state-of-the-art approaches in terms of overall accuracy, as supported by the research findings.

## Introduction

Advances in sensor technology have led to a surge of interest in recognizing human activities based on sensor data, owing to its wide-ranging applications in everyday life, such as medical care, movement analysis, intelligent monitoring systems, and smart homes. Human activity recognition (HAR) aims to study the details of human behavior in order to understand and predict specific actions. Behavior data can be obtained through various means, including accelerometers, infrared sensors, RFID, and video recordings. Currently, HAR can be categorized into two main classes: video-based and sensor-based HAR. Video-based HAR systems primarily rely on cameras to capture videos and images, utilizing computer vision technology to identify human actions and behaviors. While these techniques often yield satisfactory results, they are susceptible to environmental factors such as lighting conditions, occlusion, and privacy concerns. In contrast, sensor-based systems employ environmental or wearable sensors to determine human actions^1^. These sensors are commonly embedded in smart devices like smartphones and smartwatches. Given the ubiquity and indispensability of such devices in our daily lives, sensor-based approaches offer an immediate solution for HAR research^2,3^. The adoption of sensor-based HAR holds immense potential across numerous practical domains. For instance, it can be employed to develop advanced movement tracking systems in healthcare settings^4^, benefiting elderly individuals and disabled persons. Furthermore, it can facilitate automatic interpretation of player actions in sports^5^, enabling more streamlined analysis. Additionally, sensor-based HAR enables user identification and verification in surveillance systems by analyzing gait characteristics^6^. Lastly, it contributes to human-robot interactions through gesture recognition^7^. Harnessing the power of sensor-based HAR can bring significant advantages to these diverse sectors.

The utilization of wearable sensors in HAR has traditionally presented a complex challenge due to the classification of time-series data with multiple variables. A crucial aspect of overcoming this challenge lies in the extraction of relevant features, which can be achieved by employing mathematical methods in both the temporal and spectral domains^8^. While conventional machine learning (ML) algorithms like Naive Bayes, decision trees, and support vector machines have been successful in categorizing various human behaviors^9^, manual feature extraction requires specialized knowledge or expertise, limiting its practicality. Consequently, the use of mathematical methods for learning fails to capture distinct characteristics that can effectively differentiate complex actions. Fortunately, the introduction of convolutional layers in deep learning (DL) models has revolutionized the field by automating the feature extraction process^2^. This breakthrough empowers HAR with the capabilities of DL techniques, opening new possibilities for advancement in the field.

The convolutional neural network (CNN) model is known for its local connectivity and weight distribution mechanisms, resulting in a reduced number of parameters and faster training. Consequently, numerous studies have been published on sensor-based HAR utilizing CNN^10,11^. The effectiveness of CNN in extracting features and achieving accuracy is influenced by the depth and width of the network. A typical CNN comprises convolutional layers and pooling layers, which play a critical role in extracting feature maps essential for categorization. However, not all feature maps contribute significantly to accurately identifying targeted actions. CNN excels in capturing spatial representation from sensor data, while Recurrent Neural Networks (RNN) excel in capturing temporal representation. Therefore, combining CNN and RNN allows for a comprehensive representation of spatial and temporal features from sensor input. In a previous work by Ordonez et al.^12^, both CNN and RNN were employed for HAR. To further enhance the effectiveness of HAR, it is recommended to prioritize valuable feature maps while suppressing unreliable ones. This is addressed by the squeeze-and-excitation (SE) block^13^, which acts as a channel-attention mechanism. The SE block recalibrates each feature map by assigning a weight proportional to its significance in the identification process. Zhongkai et al.^2^ report the implementation of the SE block in CNN and/or RNN models, resulting in an improved efficacy of HAR.

The existing literature provides valuable inspiration for understanding how individual actions occur in spatial and temporal aspects. By leveraging this knowledge, we can analyze data from wearable sensors using abstract features to identify human behaviors. In this study, we propose a novel approach called ResNet-BiGRU-SE, which combines a hybrid CNN with a channel attention system, to recognize human activities based on sensor data. We conducted multiple experiments using different standard datasets for HAR to assess the effectiveness of our model. Our hybrid model surpasses previous DL models in terms of accuracy, as evidenced by its performance on evaluation metrics. Therefore, this study emphasizes the following key contributions:

1. We developed a hybrid CNN embedded with a channel attention mechanism, called ResNet-BiGRU-SE, to extract deep spatio-temporal features hierarchically and distinguish human activities in daily living.
2. Various CNN architectures have been employed as the underlying models for sensor-based HAR. To evaluate the performance of the ResNet-BiGRU-SE model, we compared its effectiveness with that of other CNN-based models on the HAR dataset. Additionally, we conducted a comparative analysis between our proposed approach and state-of-the-art models using three benchmark HAR datasets (UCI-HAR, WISDM, and IM-WSHA) for a fair assessment.

The remaining sections of the study are arranged as follows: Section “Related works” explores the research on sensor-based HAR based on DL and current frameworks; Section “Research methodology” describes the hybrid DL framework presented in this study for sensor-based HAR; and Section “Experiments and results” describes the experimental setup and provides experimental findings. This section also contains an analysis of the experimental outcomes. Section “Discussion” concludes the study and addresses future employment.

## Related works

HAR poses challenges as a time series classification problem, involving the prediction of an individual’s movements using sensory input. Typically, it necessitates extensive domain knowledge and signal processing techniques to extract appropriate features from raw data that align with a machine learning algorithm. DL methods, such as CNNs and Long Short-Term Memory Neural Networks (LSTMs), have demonstrated their effectiveness by automatically learning relevant features from raw sensory input, thereby achieving state-of-the-art performance^14,15^.

HAR aims to collect and recognize real-world actions performed by individuals or groups while considering the surrounding environmental factors. This field holds significant promise in the study of Human-Computer Interaction^16,17^ as it has the potential to revolutionize how humans interact with technology in the present era. The objectives of HAR can be categorized into five main areas: identifying fundamental movements, detecting everyday motions, recognizing unique events, forecasting caloric expenditure, and performing individual biometric recognition^18^. To achieve these goals, a variety of sensors can be utilized, including environmental sensors and wearable video cameras. In practice, wearable sensors often take the form of smartphones or sensors integrated into wearable devices.

While camera sensors can provide unique information not obtainable from other sensor types, they come with certain drawbacks. Camera-based systems require constant monitoring of individuals, resulting in the need for significant storage capacity and computational capabilities. Additionally, continuous surveillance by camera systems may lead to discomfort or unease among individuals^19^. An example of a camera-based indoor human motion tracking system is presented by Zhou et al.^20^, showcasing continuous video monitoring and advanced video processing capabilities. Another benefit of camera sensors is their ability to provide accurate data for human motion identification systems.

Ambient sensors offer the ability to monitor and record an individual’s interactions with their environment. In the experimental context of Zhan et al.’s study^21^, wireless Bluetooth acceleration and gyroscope sensors were employed to capture situational components and demonstrate their usage. Furthermore, room-side wired microphone arrays were utilized to detect ambient sound, while Reed switches were placed on doors, drawers, and shelves to detect their operation and generate contextual information. However, it should be noted that environmental sensors are limited to specific conditions and architectural configurations, rendering the HAR system non-universal. A well-designed and trained HAR system cannot be directly applied to a different environmental setting. Additionally, the implementation cost associated with these sensors tends to be relatively high.

Wearable technologies worn on the human body have the capability to recognize the physical aspects and characteristics of individuals’ everyday tasks. Inertial sensors such as accelerometers and gyroscopes, along with GPS and magnetic field sensors, are commonly used in applications for action identification. In specific studies, action identification is achieved by utilizing one or more accelerometers attached to various regions of the human body. Dong and Biawas^22^ introduced a wearable sensor network designed for HAR. Additionally, Curone et al.^23^ utilize a tri-axial accelerometer worn on the body for action recognition.

Given the significant advancements made by DL across various ML applications, and considering the inherent multi-class nature of DL techniques, our systematic review begins with a concise overview of DL for human activity detection. Wang et al.^24^ conducted a comprehensive review of 56 publications from 2011 that utilized DL techniques, including deep neural networks, CNNs, RNNs, auto-encoders, and limited Boltzmann machines, for sensor-based HAR. They found that no single model outperforms all others in every scenario, emphasizing the importance of selecting a model based on the specific application requirements. Additionally, they compared three benchmark datasets for HAR: the Opportunity dataset^25^, the Skoda dataset^26^, and the UCI-HAR dataset^27^ (collected using smartphones with multiple inertial measurement units). Among these datasets, they identified studies^12,28–30^ as representing the state-of-the-art in DL for HAR utilizing inertial measurement units (IMUs).

Sophisticated HAR models benefit from complex and deeper structures, leading to improved accuracy compared to previous feature learning methods. These models utilize CNNs for automatic feature extraction. In the context of object identification, the CNN feature extractor is often referred to as the backbone. This term emphasizes that the architecture of the feature extractor and the overall model construction are evaluated separately and independently.

Instead of relying on basic models, researchers have developed sophisticated backbone models to enhance performance. Dong et al.^31^ introduced a combination of Hierarchical Cross-Filtering (HCF) and an inception module. Long et al.^32^ proposed a method of independently learning large-scale and small-scale networks and subsequently joining them. This approach incorporates two different sizes of residual blocks as crucial components. Tuncer et al.^33^ suggested utilizing a ResNet structure with multiple layers as feature extractors, with the extracted features cascaded to serve as the backbone. Ronald et al.^34^ presented the iSPLInception backbone, which is based on Inception-ResNet and utilizes a multichannel-residual hybrid architecture for HAR research. Mehmood et al.^35^ employed DenseNet as the backbone and leveraged dense connections for HAR purposes.

## Research methodology

**Figure 1:**
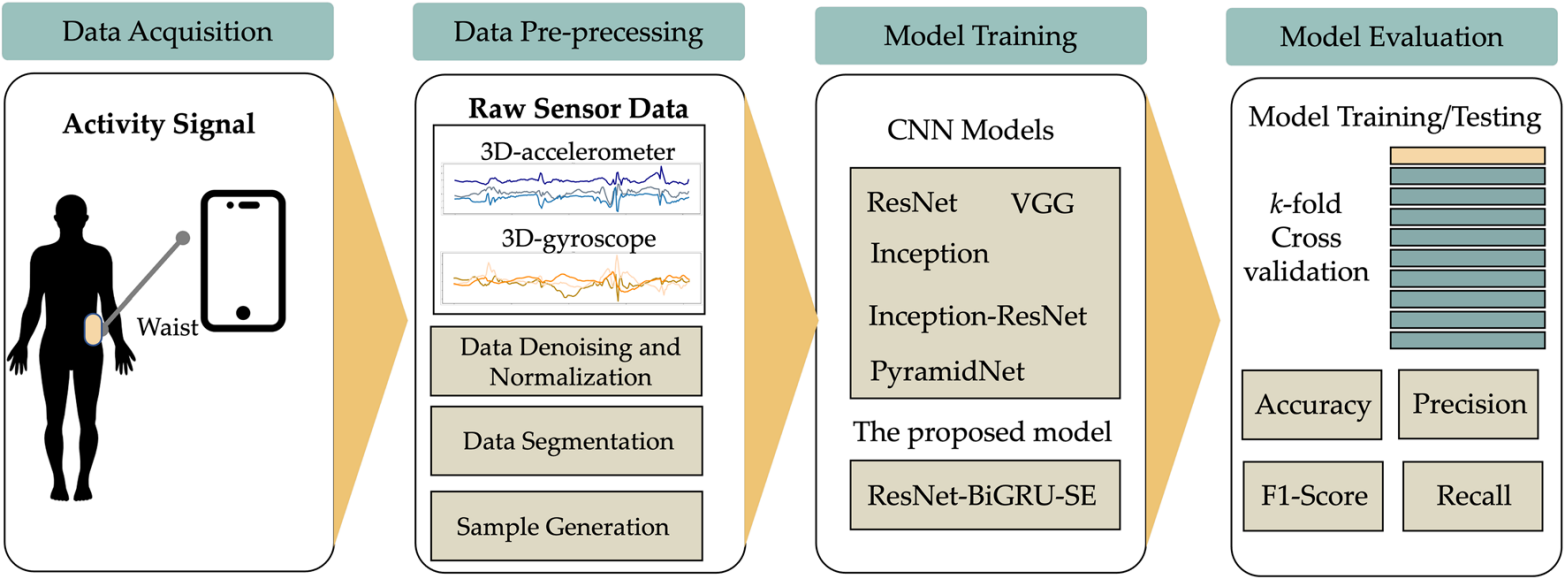
HAR workflow employed in this study.

This research investigated sensor-based HARs using DL techniques to extract abstract characteristics from raw sensor data. As shown in Fig. 1, the explored HAR framework consists of four key process steps: data acquisition, data pre-processing, model training, and model assessment.

### Data acquisition

This section highlights the HAR datasets utilized in the evaluation of this study. For assessment purposes, three public datasets were included: UCI-HAR, WISDM, and IM-WSHA. These datasets consist of inertial data collected from smartphone sensors, with each dataset capturing information from a group of individuals as they performed their daily activities. Table 1 provides a comprehensive comparison of the three benchmark datasets used in this study.

#### UCI-HAR dataset

This paper utilizes the ”UCI Human Activity Recognition Using Smartphone Dataset (UCI-HAR)”^27^ as the public activity dataset for the proposed approach. The UCI-HAR dataset comprises action data collected from a diverse group of 30 individuals with varying ages (19 to 48 years), genders, heights, and weights. Participants wore a smartphone at their waist position while performing everyday activities. Each individual engaged in six different activities. The smartphone’s tri-axial accelerometer and gyroscope captured sensor data during the execution of these six predetermined tasks. Data on triaxial linear acceleration and angular velocity were collected at a consistent rate of 50 Hz.

#### WISDM dataset

The WISDM dataset^36^ serves as a fundamental HAR dataset derived from the Wireless Sensor Data Mining Laboratory. It consists of 1,098,207 samples and captures the action recognition patterns of 36 individuals, encompassing activities such as strolling and seating. The data was collected by participants who carried an Android smartphone in their front leg pocket, utilizing the device’s built-in accelerometer sensor at a sampling rate of 20 Hz.

#### IM-WSHA dataset

The IM-Wearable Smart Home Activities (IM-WSHA) dataset^37^ is a comprehensive collection of signal data specifically designed to serve as a standard dataset for HAR. This dataset features three wearable Inertial Measurement Unit (IMU) sensors that capture three-axis accelerometer, gyroscope, and magnetometer data. The sampling frequency of the dataset is 100 Hz. To accurately capture individuals’ movement patterns during their daily activities, the IMU sensors were strategically positioned on different body parts, namely the thorax, femur, and wrist. The study involved ten participants, with an equal distribution of males and females, who performed a total of eleven distinct physical tasks within an indoor environment. These tasks encompassed various common activities such as walking, exercising, cooking, drinking, talking on the phone, doing laundry, watching television, studying, brushing hair, using a laptop, and vacuum-cleaning.

**Table 1:** A detailed comparison of three benchmark datasets used in this study.

Dataset	Sensors	Sampling rate	Activities	Subjects
UCI-HAR	Accelerometer & Gyroscope (smartphone)	50 Hz	Normal walking, walking upstairs, walking downstairs, sitting, standing, and laying	30
WISDM	Accelerometer (smartphone)	20 Hz	Normal walking, jogging, ascending, descending, sitting, and standing	36
IM-WSHA	3-IMUs	100 Hz	Normal walking, exercising, cooking, drinking, phone conversation, ironing, watching TV, reading a book, brushing hair, using the computer, and vacuum-cleaning	10

### Data pre-processing

The acquired raw data from sensors often contains measurement noise and additional unforeseen noise caused by the participant’s dynamic movements during data collection. The presence of noise in the signal distorts the usable information it carries. Therefore, it becomes crucial to reduce the influence of noise and extract valuable information from the signal for further processing. Common filtering techniques used to address this issue include mean, Low-pass, and Wavelet filtering^38,39^. In our work, we employed a third-order low-pass Butterworth filter with a cutoff frequency of 20 Hz across all three dimensions of the accelerometer, gyroscope, and magnetometer sensors for effective signal denoising. This choice of filter parameters is suitable for recording human motion since the energy content below 15 Hz accounts for 99.9% of the signal, making it an appropriate resolution.

Once the noise was removed, the filtered sensor data underwent a transformation to prepare them for further analysis. In this phase, a Min-Max normalization approach was employed to adjust each dataset’s values within the range of [0, 1]. This normalization is advantageous for learning techniques aiming to assess the effects of various factors.

During the data segmentation phase, the normalized data from all sensors is divided into equal-sized portions using fixed-size sliding windows. In this study, we chose a sliding window of 2.56 seconds, which resulted in sequences of sensory data with a specific length. These segmented portions are then used for model training.

### The proposed hybrid convolutional neural network

**Figure 2:**
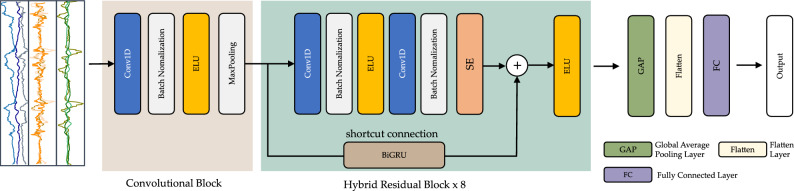
Detailed and unrolled architecture of the proposed hybrid convolutional neural network model.

This research proposes an effective biometric recognition model called ResNet-BiGRU-SE for utilizing motion signal data captured from smartphone sensors. The proposed method automatically generates identifying characteristics based on the sensor data inputs. ResNet-BiGRU-SE consists of a convolutional block and eight hybrid residual blocks, which extract standard spatial features. The model also includes a global average-pooling (GAP) layer, a flattened layer, and a fully connected layer, as illustrated in Fig. 2.

#### Convolutional block

CNNs typically employ a predefined set of elements and are commonly utilized for supervised learning. In these neural networks, each neuron is connected to every other neuron in the subsequent layers. The activation function of the neural network converts the input value of the neurons into their output value. The effectiveness of the activation function is influenced by two important factors: sparsity and the neural network’s ability to handle reduced gradient flow to its lower layers^40^. In CNNs, pooling is often used for dimension reduction. Both maximum and average pooling functions, referred to as max-pooling and average-pooling, are commonly utilized.

In this study, we utilized a convolutional block (ConvB) to process the raw sensor data and extract low-level features. The ConvB, as depicted in Fig. 2, consists of four layers: 1D-convolutional (Conv1D), batch normalization (BN), exponential linear unit (ELU), and max-pooling (MP). Conv1D employs multiple trainable convolutional kernels to capture different features, generating a feature map for each kernel. The BN layer is employed to stabilize and accelerate the training process, while the ELU layer enhances the model’s expressive capability. Additionally, the MP layer is used to reduce the size of the feature map while retaining the most significant characteristics.

#### Structure of gated recurrent unit

**Figure 3:**
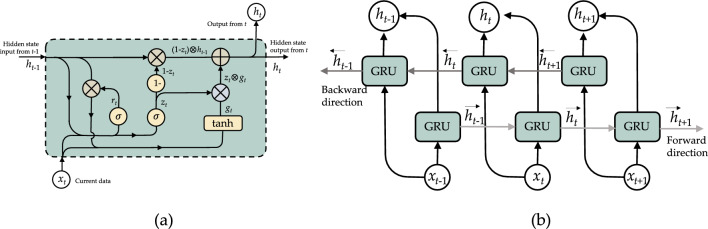
Structure of Bidirectional Gated Recurrent Unit: (**a**) GRU cell and (**b**) unroll BiGRU.

Gate recurrent unite (GRU) was developed as a new RNN-based approach to prevent the exploding/vanishing gradient issue; nevertheless, the design’s memory cells result in a higher memory consumption^41^. The GRU is a straightforward variation of the LSTM in which individual memory cells are omitted from its design^42^. As seen in Fig. 3a, a GRU’s network has an update and a reset gate that handles the update level of each concealed state, i.e., it determines which data must flow to the next stage and which does not. GRU computes the hidden state $h_t$ at time *t* based on the output of the update gate $z_t$, the reset gate $r_t$, and the current input $x_t$. The prior hidden state $h_{t-1}$ is determined as follows:

1 $$\begin{aligned} z_t= & {} s(W_zx_t \oplus U_zH_{t-1}) \end{aligned}$$

2 $$\begin{aligned} r_t= & {} s(W_rx_t \oplus U_rH_{t-1}) \end{aligned}$$

3 $$\begin{aligned} g_t= & {} \tanh (W_gx_t \oplus U_g(r_t \otimes H_{t-1})) \end{aligned}$$

4 $$\begin{aligned} h_t= & {} ((1 - z_t) \otimes h_{t-1} \oplus (z_t \otimes g_t)) \end{aligned}$$

where $\sigma$ is a sigmoid function and $\oplus$ is an elementary addition operation, and $\otimes$ is an elementary multiplication operation.

Schuster and Paliwal^43^ introduced a bidirectional RNN (BiRNN) in 1997 in order to address the drawback of a conventional (unidirectional) RNN. In addition to the present input, the output at a given period also incorporates past and future data. This is performed by concurrently training the network in the forward and reverse directions. A normal RNN does this by dividing its neurons into a portion responsible for the forward direction and a portion responsible for the reverse direction. Positive neuron output is not linked to negative neuron output, and vice versa. This results in the general structure depicted in Fig. 3b. The relevant computations are shown in the following equations:

5 $$\begin{aligned} \overrightarrow{\mathrm{h_t}} = GRU(x_t, \overrightarrow{\textrm{h}}_{t-1}) \end{aligned}$$

6 $$\begin{aligned} \overleftarrow{\mathrm{h_t}} = GRU(x_t, \overleftarrow{\textrm{h}}_{t-1}) \end{aligned}$$

7 $$\begin{aligned} h_t = [\overrightarrow{\textrm{h}}_{t}, \overleftarrow{\textrm{h}}_{t}] \end{aligned}$$

#### Hybrid residual block

Commonly, simple DL algorithms employ convolution layers followed by fully connected layers for classification tasks, without incorporating shortcut connections. These architectures are known as sequential networks, where each layer passes data to the next layer. However, as the size of the sequential network increases, a challenge arises in the form of vanishing or exploding gradients. This can pose difficulties for the effective training of such networks.

To overcome this problem, ResNet utilizes residual blocks, which allow for skip connections between blocks of convolutional layers. These skip connections enhance gradient propagation and facilitate the training of increasingly deeper CNNs, mitigating the issue of gradient vanishing. A residual layer can be represented as follows:

8 $$\begin{aligned} \text {ELU}(x) = {\left\{ \begin{array}{ll} x &{} \quad \text {if } x \ge 0\\ \alpha (e^x - 1) &{} \quad \text {if } x < 0 \end{array}\right. } \end{aligned}$$

9 $$\begin{aligned} R(x) = \text {ELU}(x + f(x)) \end{aligned}$$

Where *x* denotes the input, *f*(*x*) denotes the layer’s output, ELU(*x*) denotes the exponential linear unit function, and *R*(*x*) denotes the residual block’s output. The residual element *f*(*x*) is generated in this block as two consecutive repetitions of a trio of operational processes: convolution with a filter of size 3$\times$1, batch normalization, and ELU activation. The *f*(*x*) feature map is then concatenated with the input *x*, and the ELU activation function is then applied to the combined characteristics.

**Figure 4:**
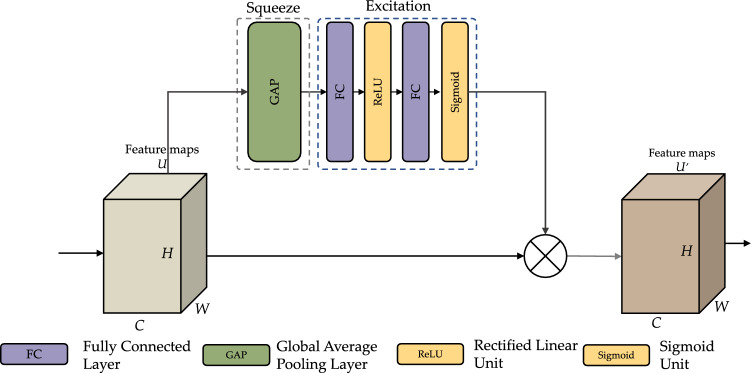
The residual block with SE module.

In order to extract hybrid features hierarchically by incorporating both spatio-temporal and channel-wise data, we introduced the SEResidual block based on previous work by Muqeet et al.^44^. As depicted in Fig. 4, this residual block consists of Conv1D layers, BN layers, ELU layers, SE modules, and shortcut connections with BiGRU. The inclusion of SE modules enhances the model’s representational capacity by incorporating channel attention.

Figure 4 illustrates the construction of a SE component. After a convolution process, several feature maps are compiled. Nevertheless, specific feature maps could include duplicated data. The SE module performs feature recalibration to improve the discriminative information and disable the less valuable aspects. This module has two primary phases: squeezing and excitation. The exponential linear unit function and *R*(*x*) is the residual block’s output. The residual element *f*(*x*) is generated in this block as two consecutive repetitions of a trio of operational processes: convolution with a filter of size 3$\times$1, batch normalization, and ELU activation. The *f*(*x*) feature map is then concatenated with the input *x*. The ELU activation function is then applied to the combined characteristics.

Initially, the squeeze process comprises all information related to the channels. H$\times$W is the size of the feature map C$\times$H$\times$W that corresponds to one channel in U. Utilizing channel descriptor function, including global average pooling (GAP), feature maps for each channel are compressed into 1$\times$1 feature map^45^. During this step, a scalar value reflecting a global channel is established. The procedure indicated by Eq. (10), where $U_c(i, j)$ is a feature map relating to channel *c* after the convolution layer has been applied to *X*. $F_{squeeze}$ is the channel descriptor function, and GAP was employed in this investigation.

10 $$\begin{aligned} Z_c = F_{squeeze}(U_c) = \frac{1}{H \times W} \sum _{i=0}^{H} \sum _{j=0}^{W}U_c(i, j) \end{aligned}$$

The channel-wise dependencies are then examined in the excitation stage utilizing the descriptor for each channel acquired in the squeeze stage. Fully-connected (FC) layers and nonlinear functions could accomplish this goal. Equation (11) describes the excitation stage, where *z* is the result acquired by squeezing, $W_i$ are the *i*th FC layers, is the sigmoid function, and $F_{excite}$ is the excitation mechanism. According to the sigmoid, the resulting value of the excitation stage is between 0 and 1 and might even be employed as a calibration weight. The current feature map U is multiplied by the newly derived weight *s*. The design of the squeeze and excitation stages in the SE block is shown in Fig. 4, along with the operation of the SE component implemented in this investigation.

11 $$\begin{aligned} s = F_{excite}(z, W) = \sigma (g(z, W)) = \sigma (W_2 \text {ReLU}(W_1z)) \end{aligned}$$

In order to deploy the activations to the side path network, the final step needs reconfiguring the output *U*, where $X = [x_1, x_2,..., x_n]$. $s_nU_n$ is the channel-wise multiplication of the scalar sn by the feature map. This procedure supplies adjustable weights to the feature channels that are the basis of the SE block^46^.

### Hyperparameters

The DL process relies on the configuration of hyperparameters, which govern the learning procedure. In the case of the ResNet-BiGRU-SE model, the following hyperparameters were utilized: (1) learning rate ($\alpha$), (2) epochs, (3) batch size, (4) optimization method, and (5) loss function. Initially, the learning rate $\alpha$ was set to 0.001. The training process involved 200 epochs and used batches of size 128. If the validation loss did not improve for 30 consecutive epochs, a predefined function was triggered to stop the training early. After six additional epochs, the ResNet-BiGRU-SE model’s learning rate was adjusted to 75% of its initial value, as the accuracy did not improve during the verification phase. To minimize errors, the Adam optimization algorithm^47^ was employed, with the following parameters: $\beta _1$ = 0.9, $\beta _2$ = 0.999, and $\epsilon$ = 1 $\times$ $10^{-8}$. For error identification, the categorical cross-entropy function^48^ was utilized, as it has demonstrated superior performance compared to classification and mean square error metrics.

### Cross validation method

**Figure 5:**
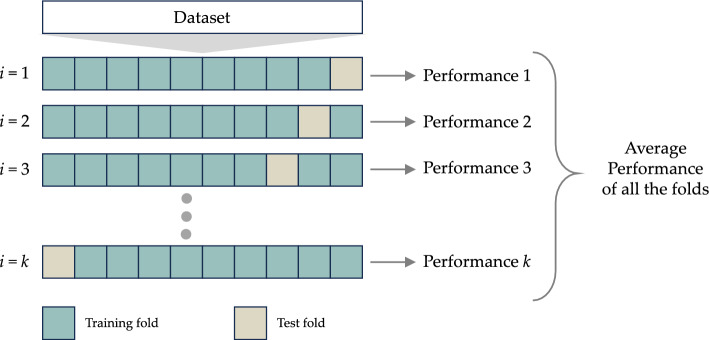
*k*-CV technique.

The k-fold cross-validation (*k*-CV) technique is a valuable method for estimating the performance of a classification model using multiple data subsets^49^. This approach involves randomly dividing a dataset, obtained from either a single individual or multiple participants, into *k* non-overlapping subsets of approximately equal size. Each subset is then used to evaluate the classification model trained on the remaining *k* - 1 subsets. The overall effectiveness of the model is determined by computing the mean value of performance measures such as accuracy, precision, recall, and F-measure, obtained from the *k*-CV^50^. It’s worth noting that this approach can be computationally demanding, particularly when dealing with large sample sizes or high values of *k*. In this study, we applied the *k*-CV technique with *k* set to 5, as depicted in Fig. 5, to assess the performance of the models.

### Performance measurement

In order to evaluate the effectiveness of the proposed DL model, we employed a 5-CV procedure. This technique enables us to comprehensively assess the model’s performance using four widely-used evaluation metrics: accuracy, precision, recall, and F-measure. The mathematical equations representing these four assessment indicators are provided below:

12 $$\begin{aligned} Accuracy = \frac{TP + TN}{TP + TN + FP + FN} \end{aligned}$$

13 $$\begin{aligned} Precision = \frac{TP}{TP + FP} \end{aligned}$$

14 $$\begin{aligned} Recall = \frac{TP}{TP + FN} \end{aligned}$$

15 $$\begin{aligned} F-measure = 2 \times \frac{Precision \times Recall}{Precision + Recall} \end{aligned}$$

The four measures discussed in this context are commonly employed to evaluate the effectiveness of sensor-based HAR. In this context, recognition refers to accurately identifying a specific category, known as true positive (TP), while correctly identifying all other categories as true negatives (TN). Misclassifying sensor data into another category results in a false positive (FP) identification. Likewise, misclassifying action sensor data from another category as belonging to the considered category leads to a false negative (FN) understanding of that category. The pseudo-code for the HAR algorithm used in this study is described in Algorithm 1.
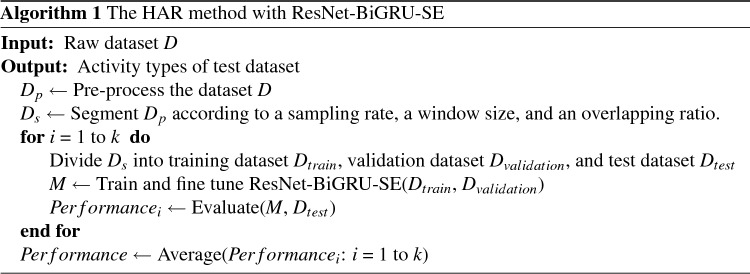


## Experiments and results

In this section, we present the studies conducted to determine the most efficient CNN models for sensor-based HAR. Our research focused on three benchmark smartphone sensing datasets, namely UCI-HAR, WISDM, and IM-WSHA datasets, which are commonly used for HAR tasks. The performance of the DL models was evaluated using accuracy and F-measure, which are widely recognized metrics for assessing model effectiveness in HAR applications.

In the investigation, we compared the CNN backbone models VGG16^51^, ResNet18^52^, PyramidNet18^53^, Inception-V3^54^, Xception^55^, and Inception-ResNet^34^. These models were presented as a solution to the issue of image recognition; consequently, we reconstructed the framework of these models for HAR. Furthermore, the identification capabilities of CNN models and our suggested model are compared.

### Experiment setting

This research utilized Google Colab Pro+ with a Tesla V100-SXM2-16GB graphics processor module to accelerate the training of DL models. The ResNet-BiGRU-SE and other primary DL models were developed in a Python library with TensorFlow and CUDA backends. These studies focused on the following Python libraries:

- Numpy and Pandas were used for managing data while retrieving, processing, and analyzing sensor data.
- Matplotlib and Seaborn were applied for charting and presenting the results of data exploration and model evaluation.
- Scikit-learn (Sklearn) was utilized as a module for sampling and data production in investigations.
- TensorFlow, Keras, and TensorBoard were operated to produce and train models using DL.

### Experimental results

In this study, we assessed the proposed framework by comparing it to baseline DL algorithms using three publicly available datasets: UCI-HAR, WISDM, and IM-WSHA. The following subsections present the experimental findings of these DL methods trained on smartphone sensing data from these benchmark datasets.

**Table 2:** Recognition performance of DL models on the UCI-HAR dataset.

Model	Parameter	Recognition performance		
		Accuracy	Loss	F-measure
Inception-ResNet	44,424,486	98.64% (±0.333%)	0.05 (±0.019)	98.72% (±0.315%)
Inception	14,286,246	98.44% (±0.972%)	0.05 (±0.029)	98.53% (±0.912%)
Xception	20,699,902	98.95% (±0.281%)	0.05 (±0.027)	99.01% (±0.272%)
VGG11	28,275,078	97.28% (±1.337%)	0.09 (±0.023)	97.45% (±1.254%)
VGG13	28,336,710	98.07% (±0.570%)	0.07 (±0.023)	98.20% (±0.531%)
VGG16	30,107,462	97.15% (±1.292%)	0.09 (±0.025)	97.28% (±1.250%)
VGG19	31,878,214	95.30% (±2.001%)	0.13 (±0.055)	95.49% (±2.063%)
ResNet16	456,262	98.37% (±1.332%)	0.08 (±0.077)	98.47% (±1.247%)
ResNet18	3,853,254	97.91% (±1.013%)	0.15 (±0.149)	97.99% (±1.040%)
ResNet34	7,238,726	96.31% (±0.921%)	0.11 (±0.044)	96.53% (±0.870%)
PyramidNet18	406,966	97.74% (±0.707%)	0.10 (±0.032)	97.43% (±0.781%)
PyramidNet34	796,414	92.09% (±13.159%)	0.76 (±1.408)	91.76% (±13.984%)
PyramidNet50	1,476,142	98.02% (±1.743%)	0.09 (±0.091)	98.13% (±1.652%)
PyramidNet101	2,503,629	98.17% (±0.342%)	0.24 (±0.329)	98.28% (±0.327%)
ResNet-BiGRU-SE	127,814	98.92% (±0.124%)	0.04 (±0.006)	98.99% (±0.119%)

In the first experiment, we evaluated the performance of the proposed ResNet-BiGRU-SE model on the UCI-HAR dataset. The results are summarized in Table 2. The findings indicate that the proposed model outperforms other CNN models, achieving an impressive average accuracy of 98.92% and an F-measure of 98.99%. It is noteworthy that the proposed model has a relatively small number of training parameters, with only 127,814 values. This demonstrates its efficiency despite its compact design.

**Table 3:** Recognition performance of DL models on the WISDM dataset.

Model	Parameter	Recognition performance		
		Accuracy	Loss	F-measure
Inception-ResNet	44,423,910	98.62% (±0.226%)	0.07 (±0.022)	97.90% (±0.398%)
Inception	14,285,670	98.43% (±0.318%)	0.08 (±0.017)	97.67% (±0.519%)
Xception	20,699,326	98.45% (±0.269%)	0.07 (±0.014)	97.60% (±0.476%)
VGG11	32,468,230	97.75% (±0.357%)	0.21 (±0.051)	96.63% (±0.485%)
VGG13	32,529,862	98.14% (±0.216%)	0.19 (±0.032)	97.21% (±0.369%)
VGG16	34,300,614	98.39% (±0.217%)	0.17 (±0.016)	97.55% (±0.366%)
VGG19	36,071,366	98.22% (±0.734%)	0.19 (±0.137)	97.30% (±1.062%)
ResNet16	454,918	98.59% (±0.370%)	0.09 (±0.036)	97.92% (±0.593%)
ResNet18	3,851,910	98.77% (±0.129%)	0.12 (±0.024)	98.20% (±0.311%)
ResNet34	7,237,382	96.75% (±1.696%)	0.34 (±0.231)	95.24% (±2.519%)
PyramidNet18	404,278	96.11% (±2.283%)	0.25 (±0.164)	94.10% (±3.507%)
PyramidNet34	793,726	98.36% (±0.395%)	0.10 (±0.018)	97.64% (±0.569%)
PyramidNet50	1,473,454	98.31% (±0.396%)	0.10 (±0.025)	97.41% (±0.616%)
PyramidNet101	2,500,941	97.21% (±1.870%)	0.082 (±0.049)	94.77% (±5.056%)
ResNet-BiGRU-SE	126,854	98.80% (±0.610%)	0.07 (±0.019)	98.62% (±0.536%)

The results presented in Table 3 are obtained from the second experiment conducted using the WISDM dataset. These findings demonstrate that the proposed ResNet-BiGRU-SE model outperforms other CNN models, achieving an impressive average accuracy of 98.80% and an F-measure of 98.62%. It is worth noting that despite its superior performance, the proposed model has a relatively small number of training parameters, with only 126,854 values. This highlights the efficiency of the model’s design in terms of parameter utilization.

**Table 4:** Recognition performance of DL models on the IM-WSHA dataset.

Model	Parameter	Recognition performance		
		Accuracy	Loss	F-measure
Inception-ResNet	44,433,899	90.57% (±7.562%)	0.52 (±0.399)	89.65% (±8.578%)
Inception	14,298,219	91.34% (±7.541%)	0.43 (±0.427)	91.10% (±7.581%)
Xception	20,711,875	97.09% (±1.093%)	0.12 (±0.058)	96.97% (±0.984%)
VGG11	36,687,627	95.09% (±0.452%)	0.50 (±0.106)	94.31% (±0.511%)
VGG13	36,749,259	91.24% (±6.768%)	0.50 (±0.107)	90.33% (±7.885%)
VGG16	38,520,011	92.46% (±1.078%)	0.58 (±0.311)	91.74% (±1.160%)
VGG19	40,290,763	91.40% (±1.822%)	0.71 (±0.104)	90.06% (±2.422%)
ResNet16	460,939	88.52% (±9.745%)	0.95 (±1.163)	87.42% (±10.380%)
ResNet18	3,859,851	96.96% (±0.842%)	0.15 (±0.045)	96.74% (±0.873%)
ResNet34	7,245,323	96.19% (±0.920%)	0.15 (±0.058)	95.97% (±0.989%)
PyramidNet18	415,595	94.60% (±3.094%)	0.27 (±0.234)	94.19% (±3.260%)
PyramidNet34	805,043	94.48% (±4.904%)	0.37 (±0.526)	93.35% (±6.522%)
PyramidNet50	1,486,451	96.51% (±0.366%)	0.17 (±0.016)	96.27% (±0.290%)
PyramidNet101	2,513,638	96.29% (±0.377%)	0.18 (±0.023)	96.02% (±0.361%)
ResNet-BiGRU-SE	130,859	98.45% (±0.269%)	0.07 (±0.014)	97.60% (±0.476%)

The findings from the third investigation, which utilized the IM-WSHA dataset, are summarized in Table 4. The results clearly demonstrate that the ResNet-BiGRU-SE model outperforms other CNN models, achieving a remarkable accuracy rate of 98.45% and an F-measure of 97.60%. These results highlight the superior performance of the ResNet-BiGRU-SE model in accurately classifying activities based on the IM-WSHA dataset.

## Discussion

### Comparison results with state-of-the-art models

We conducted a comprehensive comparison of our proposed model with state-of-the-art DL models in the field of sensor-based HAR. In Table 4, we compared our ResNet-BiGRU-SE network with several other DL techniques, namely 1D-CNN^56^, Bidir-LSTM^57^, CNN-LSTM^58^, SDAE^59^, and CNN-GRU^60^. Each of these models was developed in accordance with its respective study descriptions.

Notably, our suggested ResNet-BiGRU-SE model achieved an outstanding success rate of 98.92% on the UCI-HAR dataset, surpassing the performance of all the other models. The comparative results are presented in Table 5, providing a clear illustration of the superior performance of our proposed ResNet-BiGRU-SE model in comparison to the other models.

**Table 5:** Comparison results of the proposed model and previous works using UCI-HAR dataset.

Year	Model	Accuracy
2017	1D-CNN^56^	92.71%
2019	Bidir-LSTM^57^	92.67%
2020	CNN-LSTM^58^	92.13%
2020	SDAE^59^	97.15%
2022	CNN-GRU^60^	96.71%
Present	ResNet-BiGRU-SE	98.92%
	(Proposed model)	

In our evaluation using the WISDM dataset, we compared our proposed model with state-of-the-art DL algorithms for sensor-based HAR. Table 6 provides a comprehensive comparison of our ResNet-BiGRU-SE network with several other DL approaches, including LSTM^61^, CNN with statistical features^14^, U-Net^62^, and CNN-GRU^60^. Each of these models was implemented based on the descriptions provided in their respective publications.

Remarkably, our suggested ResNet-BiGRU-SE model achieved an impressive accuracy of 98.80% on the WISDM dataset, outperforming all the other models. This outstanding performance further highlights the superiority of our proposed ResNet-BiGRU-SE model in accurately classifying activities based on the WISDM dataset.

**Table 6:** Comparison results of the proposed model and previous works using WISDM dataset.

Year	Model	Accuracy
2016	LSTM^61^	95.78%
2020	CNN with statistical feature^14^	93.30%
2020	U-Net^62^	96.40%
2022	CNN-GRU^60^	97.18%
Present	ResNet-BiGRU-SE (proposed model)	98.80%

The findings from our study strongly support our hypothesis that our hybrid DL model, which combines local spatio-temporal characteristics with long-term contextual understanding, improves the comprehension of sensor data and ultimately enhances the performance of activity classification. Additionally, the results suggest that deep residual models exhibit favorable performance when applied to raw signals. However, the inclusion of BiGRU and SE modules further enhances the effectiveness of HAR for real-life human motion detection. These findings highlight the significance of incorporating both architectural enhancements and feature extraction techniques in order to achieve optimal results in HAR applications.

Table 7 provides a comprehensive evaluation of various advanced techniques on the IM-WSHA dataset. One approach utilized a reweighted genetic algorithm (GA) to combine statistical and frequency features extracted from a previous study^63^, resulting in an accuracy of 81.92%. Another approach involved the utilization of a random forest model with stochastic gradient descent (SGD) optimization, denoted as^64^, which achieved a recognition accuracy rate of 90.18% in order to enhance the effectiveness of HAR. Remarkably, when applied to the IM-WSHA dataset, the ResNet-BiGRU-SE model achieved an impressive identification accuracy of 98.45%. These results highlight the superiority of our proposed model over other advanced techniques in accurately identifying human activities based on the IM-WSHA dataset.

**Table 7:** Comparison results of the proposed model and previous works using IM-WSHA dataset.

Year	Model	Accuracy
2020	GA-based classifier^63^	81.92%
2022	Random Forest with SGD^64^	90.18%
Present	ResNet-BiGRU-SE (proposed model)	98.45%

### Influence of validation methods

Sensor-based HAR studies commonly employ three validation techniques: hold-out validation^65^, *k*-CV, and Leave-One-Subject-Out cross-validation (LOSO)^50^. Hold-out validation involves dividing the dataset into a training set (typically 70% of the data) and a test set (30% of the data). On the other hand, *k*-CV repeatedly partitions the dataset into *k* subsets for training and testing, evaluating the algorithm’s performance *k* times. LOSO validation creates a training set with *n* - *p* samples and a testing set with *p* samples, where *p* represents all data from a single subject. This approach ensures that there is no overlap between subjects in the training and testing sets.

**Figure 6:**
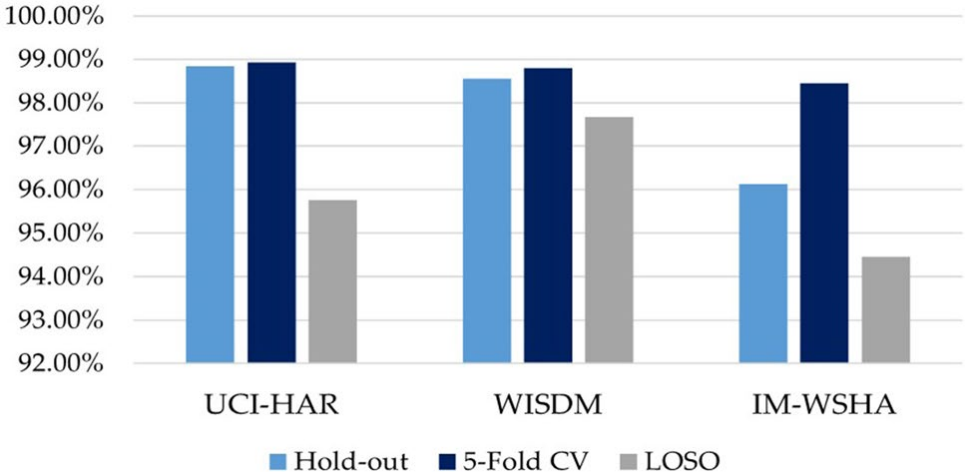
Comparative results of the proposed ResNet-BiGRU-SE conducted on different validation methods.

To assess the impact of these validation techniques, we conducted supplementary investigations using three HAR datasets: UCI-HAR, WISDM, and IM-WSHA. We evaluated the effectiveness of the ResNet-BiGRU-SE model and presented the results in Fig. 6. These evaluations allowed us to determine how different validation techniques influenced the performance of our proposed model in HAR tasks.

The findings presented in Fig. 6 clearly demonstrate the significant impact of validation approaches on the effectiveness of HAR. Among the three benchmark datasets, the ResNet-BiGRU-SE model, which utilized *k*-CV, achieved the highest levels of accuracy. However, it is important to note that this result may be influenced by the fact that the *k*-CV method does not consider scenarios where all samples come from a single study participant. This issue arises due to the time series segmentation used in the pre-processing phase. In a generalized HAR implementation, independently dividing the dataset can lead to instances where a participant’s data appears in both the training and test sets simultaneously, causing data leakage that artificially inflates the classifier’s accuracy.

On the other hand, when adopting the LOSO approach, which takes into account individual-specific data (i.e., subject labels), the accuracy of the classification model tends to decrease. The implementation of this improved assessment approach resulted in a 12% reduction in accuracy, indicating a preliminary overestimation of the results. It is important to consider these factors when selecting a validation approach to ensure accurate and reliable performance evaluation in HAR tasks.

### Misclassification

To analyze the misclassification patterns of the suggested model, we conducted a comprehensive examination of the confusion matrices generated by the ResNet-BiGRU-SE model on three different HAR datasets: UCI-HAR, WISDM, and IM-WSHA. These datasets contain activity data collected from a variety of sensor categories, as summarized in Table 1. By studying the confusion matrices, we can gain insights into the specific activities that are frequently misclassified by the model and identify potential areas for improvement.

**Figure 7:**
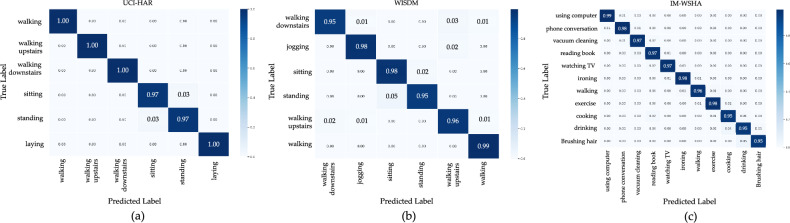
Comparison results of the proposed model and previous works using IM-WSHA dataset.

Regarding the UCI-HAR dataset, the categories of ”sitting” and ”standing” exhibited the highest frequency of misclassifications. This can be attributed to the similarity in linear acceleration patterns observed in these static actions^66^. However, the ResNet-BiGRU-SE model performed well in accurately categorizing the other four activities, as shown in Fig. 7a. Moving on to the WISDM dataset, Fig. 7b presents the confusion matrix of the model. It can be observed that the classification of ”walk upstairs” and ”walk downstairs” resulted in the highest number of errors, likely due to the contrasting nature of these two activities as different forms of physical movement. The utilization of gyroscope sensor data played a crucial role in distinguishing between these actions^67^. Lastly, the confusion matrix depicted in Fig. 7c reveals that the ResNet-BiGRU-SE model applied to the IM-WSHA dataset encountered misclassifications primarily in hand-oriented actions such as ”cooking,” ”drinking,” and ”brushing hair,” as suggested in our study. However, the model demonstrated high accuracy in classifying other diverse behaviors.

## Conclusions

This study focuses on examining the recognition performance of CNN-based classifiers with diverse topologies for sensor-based HAR. We conducted experiments using three widely-used HAR datasets: UCI-HAR, WISDM, and IM-WSHA, to thoroughly investigate the effectiveness of CNN-based models. The findings indicate that the ResNet architecture stands out as a suitable choice for HAR compared to other backbone architectures. In particular, we introduce a lightweight residual network, named ResNet-BiGRU-SE, specifically designed for sensor-based HAR. The proposed model’s efficiency was evaluated using the three datasets. By employing the 5-CV technique, our results demonstrate the superiority of our proposed model, achieving an impressive average recognition performance of 98.92% for UCI-HAR, 98.80% for WISDM, and 98.45% for IM-WSHA. Furthermore, our suggested model exhibits a reduced number of training parameters compared to previous HAR models. Moving forward, our future work aims to expand the scope of our research by including scenarios with larger populations, diverse locations, various sensor types, and activities of daily living (ADLs).

## Data Availability

The datasets used to support the findings of this study have been published online in the UCI Machine Learning Repository and other publicly accessible data sources. The UCI-HAR, WISDM, and IM-WSHA datasets can be accessed at the following link: “https://archive.ics.uci.edu/ml/datasets/human+activity+recognition+using+smartphones”, “https://archive.ics.uci.edu/ml/datasets/WISDM+Smartphone+and+Smartwatch+Activity+and+Bio-metrics+Dataset+” and “https://portals.au.edu.pk/imc/Pages/Datasets.aspx” respectively.
